# Insight into the Roles of Proline-Rich Extensin-like Receptor Protein Kinases of Bread Wheat (*Triticum aestivum* L.)

**DOI:** 10.3390/life12070941

**Published:** 2022-06-23

**Authors:** Shumayla, Venugopal Mendu, Kashmir Singh, Santosh Kumar Upadhyay

**Affiliations:** 1 Department of Botany, Panjab University, Chandigarh 160014, India; shumaylasaifi5@gmail.com; 2 Department of Plant Science and Plant Pathology, Montana State University, Bozeman, MT 59717-3140, USA; venugopal.mendu@montana.edu; 3 Department of Biotechnology, Panjab University, Chandigarh 160014, India; kashmirbio@pu.ac.in

**Keywords:** abiotic stress, biotic stress, development, evolutionary, expression, EXT motif, PERK

## Abstract

Proline-rich extensin-like receptor protein kinases (PERKs) are known for their roles in the developmental processes and stress responses of many plants. We have identified 30 *TaPERK* genes in the genome of *T. aestivum*, exploring their evolutionary and syntenic relationship and analyzing their gene and protein structures, various cis-regulatory elements, expression profiling, and interacting miRNAs. The *TaPERK* genes formed 12 homeologous groups and clustered into four phylogenetic clades. All the proteins exhibited a typical domain organization of PERK and consisted of conserved proline residue repeats and serine-proline and proline-serine repeats. Further, the tyrosine-x-tyrosine (YXY) motif was also found conserved in thirteen TaPERKs. The *cis*-regulatory elements and expression profiling under tissue developmental stages suggested their role in plant growth processes. Further, the differential expression of certain *TaPERK* genes under biotic and abiotic stress conditions suggested their involvement in defense responses as well. The interaction of *TaPERK* genes with different miRNAs further strengthened evidence for their diverse biological roles. In this study, a comprehensive analysis of obtained *TaPERK* genes was performed, enriching our knowledge of *TaPERK* genes and providing a foundation for further possible functional analyses in future studies.

## 1. Introduction

Plants are susceptible to a diverse range of environmental stimuli and have developed mechanisms for adapting to changes in their surroundings. In plants, like in other eukaryotes, a diverse range of cell surface receptor-like protein kinases (RLKs) is required for signal transduction pathways to function properly [1,2]. In response to a stimulus, these membrane-spanning proteins transfer the signal intracellularly via a signaling cascade, eliciting the appropriate cellular responses. The members of plant RLKs have a highly conserved, substrate-specific, cytoplasmic catalytic domain, known as serine/threonine kinase, while their extracellular domains are quite diverse, allowing these proteins to respond selectively to a variety of external stimuli [1,3]. Many different kinds of plant RLKs have been recognized based on the amino acid sequence motif present in their extracellular domains [4,5].

Proline-rich extensin-like receptor protein kinases (PERKs) represent an important gene family in the RLK superfamily, which is known to be involved in numerous vital functions in plants [6,7]. Similar to the other RLKs, the members of the *PERK* gene family also consist of three domains: an extracellular proline-rich domain, a transmembrane region, and an intracellular kinase domain which is responsible for catalytic activity [8,9]. The extracellular domain is densely packed with consecutive prolines, some of which form a characteristic extensin (EXT) motif with serine-proline repetitions (2–5 times), while also lacking an adjacent tyrosine-X-tyrosine (YXY) required for tyrosine mediated cross-linking [10].

The first *PERK* gene in plants was reported from *Brassica napus*, which was wound inducible and ubiquitously expressed in the vegetative and reproductive tissues of the plant [11,12]. Later on, a total of 15 *AtPERK* genes were reported in the genome of *Arabidopsis thaliana*, which consisted of variable lengths of the extracellular domain due to the occurrence of proline-rich regions in both the extracellular and cytoplasmic regions [11,13]. Out of 15, nine *AtPERK* genes exhibited significant expression in various plant parts. For instance, *AtPERK8* and *AtPERK13* showed significant expression in root hairs, while the other seven genes (*AtPERK3-7, 11* and *12*) suggested specific functions in pollen tubes [8,9]. *AtPERK13* is reported to be involved in root hair growth [6,7]; however, *AtPERK4* is associated with abscisic acid and calcium signaling during root tip development [14]. Further, it has been reported that the *AtPERKs* are also involved in the regulation of apical dominance, cell proliferation, wall deposition, reproductive tissue development and fertilization [8,14,15,16]. Additionally, the modulated expression of various *GhPERK* genes of *Gossypium hirsutum* during various abiotic stress conditions (cold, heat, drought/osmotic, and salt) revealed their role in stress response [17]. Further, *GhPERKs* also exhibited differential expression under phytohormones stress (GA, IAA, SA, and MeJA). These studies suggested numerous, diverse functions of *PERK* genes in plants. 

Despite its diverse roles in plants, an inclusive genome-wide characterization of the *PERK* gene family has been limited to only a few plant species like *Arabidopsis*, *Gossypium*, *Brassica rapa*, etc. [9,13,17]. No information is available about the *PERK* genes in bread wheat (*Triticum aestivum* L.), which is widely cultivated as an important cereal crop. The growing body of genomic data on bread wheat has enabled the inclusive exploration of numerous important gene families [18,19]. In the present study, the *TaPERK* genes of bread wheat were identified and systematically characterized in terms of their physicochemical properties, chromosomal distribution, exon/intron, protein domains and motifs, phylogeny and syntenic relationship. Further, the RNA-Seq data from five tissues with three developmental stages were analyzed to investigate their roles in plant growth and development, while the RNA-Seq data generated under heat, drought, salt, and fungal pathogen stress conditions were used to analyze their functions in stress response. The expression data of a few genes in abiotic stress conditions were validated by quantitative real-time PCR (qRT-PCR). To gain further insights into their mechanisms, interaction analyses of *TaPERKs* were performed with the known miRNAs of *T. aestivum*. These results will increase our understanding of the vital role of *TaPERK* genes and enable their functional characterization in future studies.

## 2. Results

### 2.1. Identification and Distribution of TaPERK Genes

**Figure 1:**
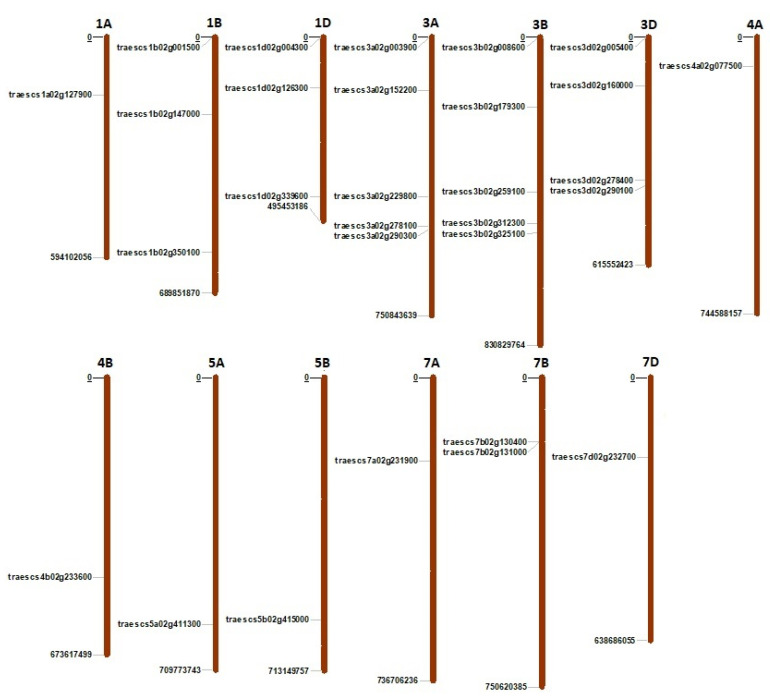
Chromosomal localization of 30 TaPERK genes. The bars represent the chromosomes. The number of the chromosome is written at the top of each bar.

A total of 30 *TaPERK* genes were identified in the genome of *T. aestivum* by extensive BLAST search using 15 PERK sequences of *Arabidopsis thaliana* and eight sequences of *Oryza sativa* (Table S1). These genes were found to be clustered into 12 homoeologous groups and were named according to the standard wheat symbolization protocol (Table S2). The majority of the *TaPERK* genes were derived from the B subgenome (40%), followed by the A (30%) and D subgenomes (27%). The *TaPERK* genes were localized on the majority of chromosomes and subgenomes, except chromosomes 2A, 2B, 2D, 4D 5D, 6A, 6B, and 6D. The maximum number of genes were located on chromosomes 3A and 3B, with five genes each, followed by four genes on the 3D chromosome. A single *TaPERK* gene was found on chromosomes 1A, 4A, 4B, 5A, 5B, 7A, and 7D. The location of one gene (TraesCSU02G104700) could not be mapped to any chromosome (Figure 1, Table S2).

### 2.2. Phylogeny and Synteny Analysis

**Figure 2:**
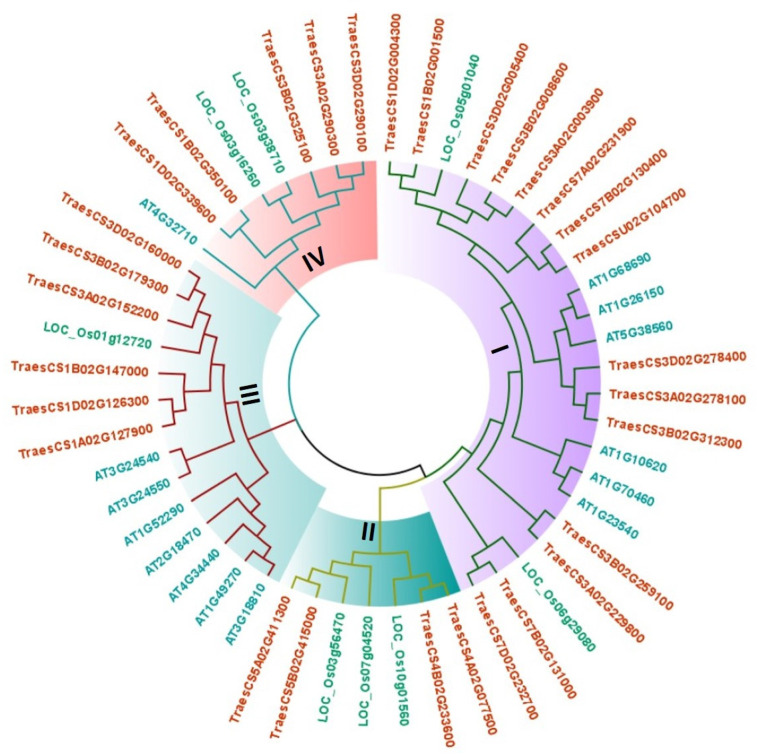
PERK proteins phylogenetic analysis. The evolutionary relationship was investigated using full-length PERK protein sequences of *A. thaliana, O. sativa*, and *T. aestivum*. The tree was constructed by the Neighbor-Joining method using the MEGA X and the bootstrap value was set to 1000. The phylogenetic tree is divided into four different distinct clades, each labeled with a different color.

The full-length PERK protein sequences of *A. thaliana*, *O. sativa* and *T. aestivum* were used for the construction of a Neighbor-Joining phylogenetic tree using the MEGA X software (Figure 2). The PERK proteins were tightly clustered into four clades which were further subdivided into sub-clades. Clade I consisted of the maximum number (44%) of the PERK proteins, followed by clade III (27%) and Clade II (15%). Clade II contained the lowest number (7) of PERK proteins. A total of 17, 6, and 2 PERK proteins of *T. aestivum, A. thaliana* and *O. sativa*, were clustered into clade I, respectively. The PERK proteins of *O. sativa and T. aestivum* were distributed among all the four clades, while clade IV lacked Arabidopsis PERK proteins. The homeologous TaPERK proteins were closely clustered in each group, for instance, TraesCS3B02G008600, TraesCS3A02G003900 and TraesCS3D02G005400. Further, the PERK proteins identified as orthologs in *T. aestivum, A. thaliana* and *O. sativa,* were also found clustered in the vicinity, such as At4G32710, At5G38560, LOC_Os03g38710, LOC_Os05g01040 were found near TraesCS3D02G160000, TraesCS3D02G278400, TraesCS3B02G325100, TraesCS1B02G001500, respectively.

**Figure 3:**
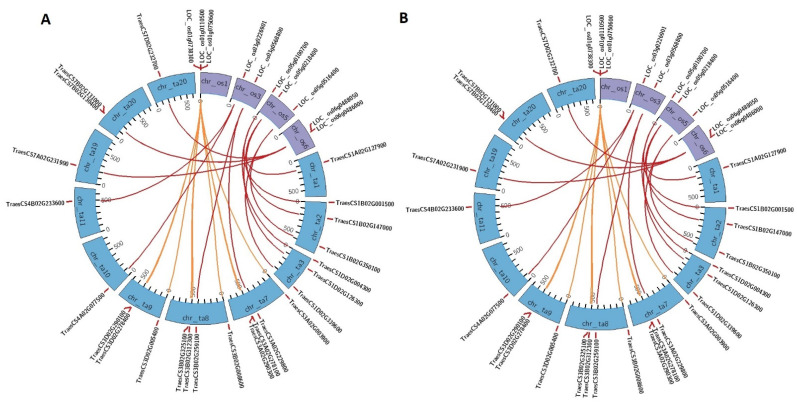
Syntenic association of *TaPERK* genes with *PERK* genes of (**A**) *A. thaliana* and (**B**) *O. sativa*. The figure was generated using the Circos 0.69-9 software. Different colors represent the chromosomes of different plant species.

Further, we performed a synteny analysis to study the orthologous relationships among the *PERK* genes of *A. thaliana, O. sativa, and T. aestivum*. A total of 13 *AtPERK* genes of *A. thaliana* exhibited close syntenic association with 25 *TaPERK* genes of *T. aestivum* (Figure 3A). However, all the *OsPERK* genes of *O. sativa* showed tight syntenic relation with 24 *TaPERK* genes of *T. aestivum* (Figure 3B). The findings revealed a tight evolutionary link among the orthologous *PERK* genes in these plant species. Furthermore, the close clustering of the orthologous PERKs of these plant species suggested significant homology among them.

### 2.3. Gene Structure Analysis

**Figure 4:**
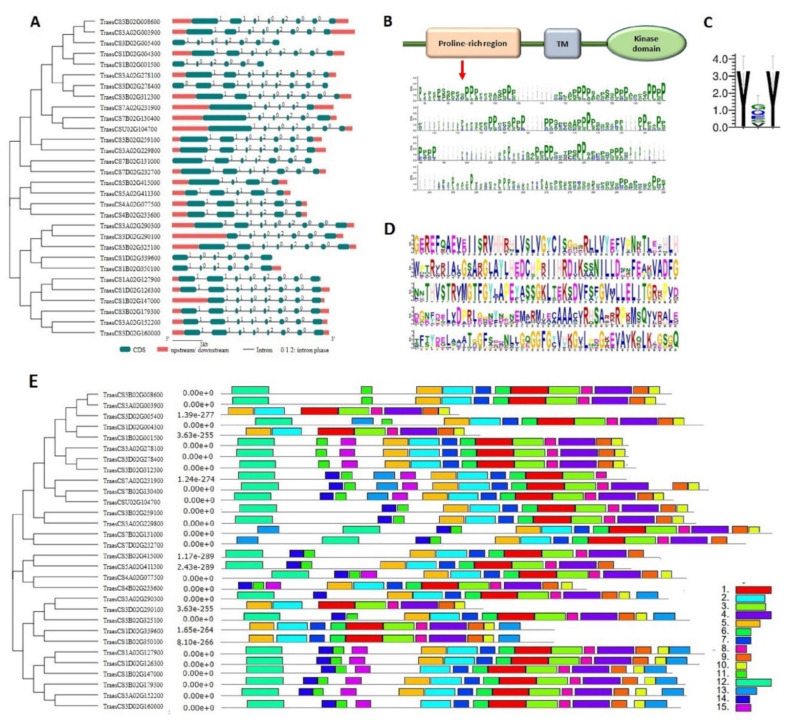
Gene and protein structure analyses. The figure represents (**A**) exon-intron architecture, with exons shown as green boxes and lines representing introns with intron phases; (**B**) the domain architecture of TaPERK proteins along with the WebLogo generated from the proline-rich region of the extracellular domain of TaPERK proteins; (**C**) Conserved YXY motif detected in 13 TaPERK proteins; (**D**) The conserved motif logos among PERK proteins of *A. thaliana*, *O. sativa,* and *T.* *aestivum*; (**E**) The schematic representation of conserved motifs of TaPERK proteins investigated using the MEME. Different conserved motifs are represented by different colored boxes. The size of the boxes indicates the length of the conserved motifs.

The gene structure of all the *TaPERK* genes was investigated for the intron phases and exon-intron organization. The number of exons ranged from five to nine in *TaPERK* genes. Around 43% of the genes consisted of eight exons, followed by 23% with seven exons. Only two genes, TraesCS5A02G411300 and TraesCS5B02G415000, consisted of five exons (Figure 4A). It was observed that the majority of *TaPERK* genes shared the conserved pattern of exon-intron arrangement across the tightly packed genes in the phylogenetic tree. For instance, TraesCS3A02G229800, TraesCS3B02G259100, TraesCS7D02G232700 and TraesCS7B02G131000 shared a similar number (seven) of exons. Among *TaPERK* genes, 44% of introns were found to be in intron phase 0, followed by 41% in phase 1, and 15% in phase 2. The *TaPERK* genes of clade I predominantly consisted of phase zero introns, which suggested their sequence conservation. Meanwhile, clade II consisted of the least number of introns in phase zero.

### 2.4. Physicochemical Properties, Domain and Motif Analysis

The peptide length of TaPERKs varied from 341 to 811 amino acid (aa) residues (Table S2). The molecular weight (MW) ranged from 37.88 kDa (TraesCS3D02G005400) to 84.78 kDa (TraesCS3B02G325100). The majority of TaPERKs consisted of a single transmembrane helix, with the exception of five proteins, TraesCS1B02G001500, TraesCS1B02G350100, TraesCS1D02G339600, TraesCS3D02G005400, and TraesCS3D02G290100, which lacked the transmembrane region. While TraesCS5B02G415000 consisted of two transmembrane helices. Furthermore, except for two proteins (TraesCS3D02G290100 and TraesCS3D02G005400) predicted to be cytoplasmic, it was hypothesized that the rest of the TaPERK proteins would be localized in the plasma membrane (Table 1). The isoelectric point (pI) varied from 5.11 to 10.06, with half of the proteins having a pI greater than 7. The grand average hydropathy (GRAVY) value varied from −0.645 (TraesCS7D02G232700) to −0.31 (TraesCS7D02G232700) (TraesCS1B02G001500) Table S2). All the members of the TaPERK family lacked the signal peptide region.

To better understand the functional role of the TaPERK family, a domain analysis was performed. TaPERK proteins were analyzed for the presence of a conserved domain using the SMART server. The results revealed the occurrence of a typical RLKs structure, i.e., a conserved cytoplasmic ser/thr kinase domain, a transmembrane region, and an extracellular region in 25 out 30 TaPERK proteins (Figure 4B). Five TaPERKs (TraesCS1B02G001500, TraesCS1B02G350100, TraesCS1D02G339600, TraesCS3D02G005400, and TraesCS3D02G290100) lacking the transmembrane region, that could be receptor-like cytoplasmic kinases. Further, a detailed analysis of protein sequences for conserved motifs was carried out using the multiple sequence alignments, WebLogos, and MEME suite server (Figure 4B–E). The multiple sequence alignment showed the conserved proline-rich region in the extracellular region of all the TaPERK protein sequences. Several densely packed consecutive proline-rich regions were found in each TaPERK protein (Figure 4B). Further, all the TaPERK proteins consisted of two to four serine-proline (SP) or proline-serine (PS) repetitions at various locations (Figure S1), which is a characteristic of the extensin (EXT) motif. Additionally, a tyrosine-X-tyrosine (YXY) motif was also found conserved in 13 out of 30 TaPERK sequences, i.e., TraesCS7B02G130400, TraesCS1B02G147000, TraesCS7A02G231900, TraesCS5B02G415000, TraesCS4A02G077500, TraesCS5A02G411300, TraesCS1A02G127900 etc. (Figure 4C). Five highly conserved regions were detected from the PERK proteins of *A. thaliana*, *O. sativa* and *T. aestivum* (Figure 4D). The MEME server identified 15 conserved motifs in TaPERK proteins (Figure 4E and Figure S2). Motifs 1 to 7 were conserved in the kinase domain region of TaPERK proteins. Motifs 1 and 2 were conserved in the ATP binding site and active site of the kinase domain region, whereas motifs 12 to 15 were conserved in the extracellular region of TaPERK proteins, which is a proline-rich region.

### 2.5. Promoter Analysis of TaPERK Genes

**Figure 5:**
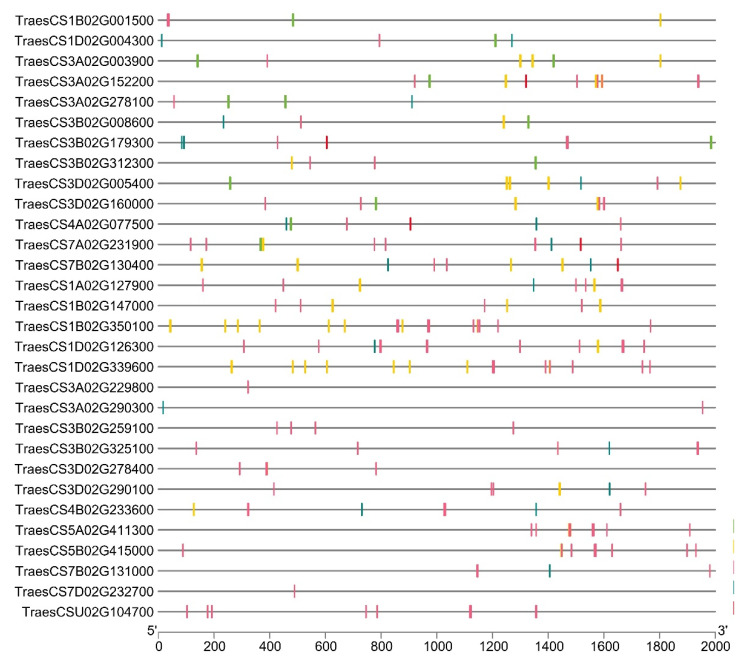
Promoter analysis. The figure represents the various *cis*-regulatory elements present in the 2 Kb upstream regions of *TaPERK* genes, and has been shown with different colored boxes. DSRE-drought stress-responsive element; ABRE-Abscisic acid-responsive elements; LRE-Low temperature-responsive element; MBS- MYB binding site involved in drought-inducibility; GRE-Gibberellin responsive element.

Comprehending gene regulation and function requires a thorough understanding of the *cis*-elements that affect gene expression through the binding of transcription factors to these elements. The *cis*-regulatory elements (CREs) in the promoter region of each *TaPERK* gene were identified in the 2 Kb upstream region from the start codon using the PlantCARE database. The identified CREs were broadly divided into defense and stress-responsive, hormone-responsive, light-responsive, etc. (Figure 5). The majority of the *TaPERK* genes promoter regions consisted of CREs related to light-responsive and hormone-responsive (Table S3). Various CREs were found in the upstream of *TaPERK* genes for different responses. For instance, meristem development (CAT-box motif) in TraesCS1B02G350100, TraesCS1B02G147000 etc., endosperm development (GCN4 motif) in TraesCS4B02G233600, TraesCS1B02G001500 etc., seed-specific regulation (RY elements) in TraesCS3B02G325100, TraesCS7B02G130400 etc., defense and stress response (TC-rich repeats and MBS) in TraesCS1B02G001500, TraesCS1D02G004300 etc., light-responsive (ATCT-motif, G-box, GA-motif, GATA-motif, GC-motif, GAG-motif, GT1-motif, Sp1, TCT-motif) in TraesCS1D02G004300, TraesCS1D02G126300, TraesCS1D02G339600, etc., low-temperature responsive elements (LTR) in TraesCS3A02G229800, TraesCS3A02G278100, etc. The cis-elements regulating the responses via different hormones were abscisic acid-responsive element (ABRE), methyl jasmonate responsive elements (CGTCA-motif and TGACG-motif) auxin (TGA-element), salicylic acid (TCA-element), and gibberellin (TATC-box and P-box motifs), were also found in the promoter regions of *TaPERK* genes such as TraesCS3A02G229800, TraesCS3A02G290300, TraesCS3A02G278100, TraesCS3B02G259100, TraesCS3D02G005400 etc., respectively. 

### 2.6. Transcriptional Profiling in Tissue Developmental Stages

**Figure 6:**
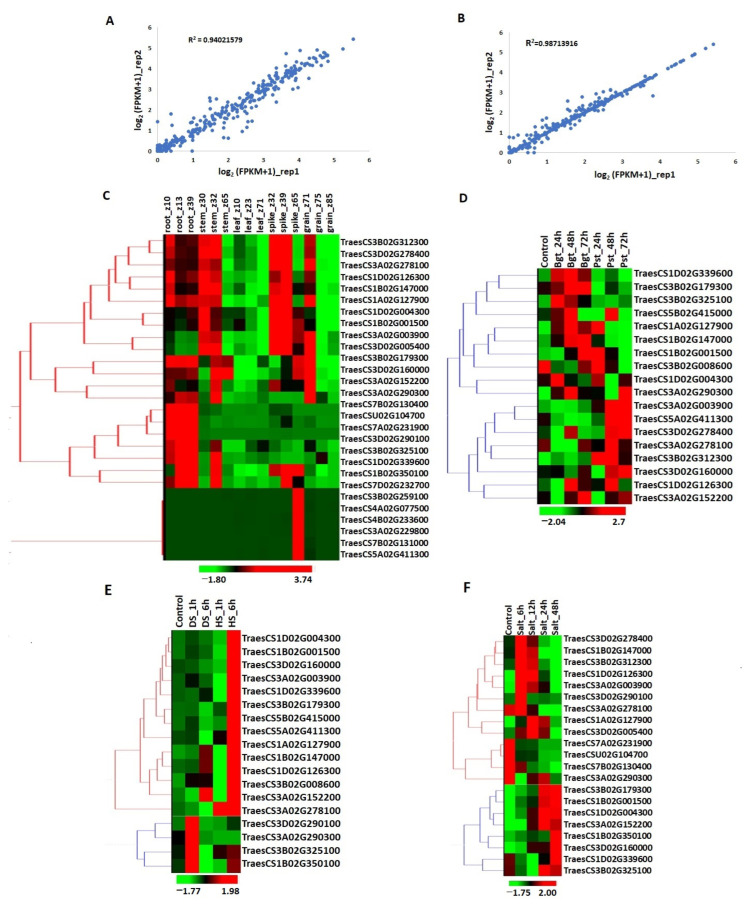
Heatmaps depicting the relative expression of *TaPERK* genes in five distinct tissues at various developmental stages and under various stress conditions. The graphs show the correlation analysis of expression data under (**A**) tissue developmental stages and (**B**) stress conditions. Heat maps were generated from high throughput RNAseq data for (**C**) five different tissues (root, stem, leaf, spike, and grain) and three development stages (in Zadoks scale), (**D**) after 24, 48, and 72 h of *Blumeria graminis* and *Puccinia striiformis* inoculation, (**E**) after 1 and 6 h of heat stress (HS), drought stress (DS), and (**F**) after 6, 12, 24 and 48 h of salt stress treatments.

To analyze the involvement of *TaPERK* genes in the growth and development of plants, transcriptional profiling during tissue developmental stages was performed using the high throughput RNA seq data. A correlation analysis was done to study the consistency of the expression data. A significant correlation (R^2^ = 0.94) was observed in replicates of the expression data (Figure 6A). During the root, stem, leaf, spike, and grain developmental stages, *TaPERK* genes sowed differential Spatio-temporal expression patterns (Figure 6C). Around 67% of *TaPERK* genes such as TraesCS3B02G312300, TraesCS3D02G278400, TraesCS3B02G179300, etc. showed higher expression in all the root developmental stages, followed by 60% genes were highly expressed in the stem_z30 and stem_z32 developmental stages, for instance, TraesCS3B02G179300, TraesCS3D02G160000 etc. However, only two genes (TraesCS3B02G179300 and TraesCS3B02G160000 exhibited significant expression in stem_z65. All the *TaPERK* genes were low expressing in all the leaf developmental stages. About 46% of genes showed maximum expression in the spike developmental stages. For example, TraesCS1D02G004300, TraesCS1B02G001500, TraesCS3D02G005400 etc. showed the highest expression in early developmental stages of spike. Interestingly, it was observed that six genes namely TraesCS3D02G290100, TraesCS4A02G077500, TraesCS4B02G233600, TraesCS3A02G229800, TraesCS5A02G411300 and TraesCS7B02G131000 were predominantly expressed in the later stage of spike development viz. spike_z65. In the grain tissue, few *TaPERK* genes showed higher expression in the initial stage of grain development, i.e., grain_z71, while in the later stages, the genes showed lower expression. For instance, TraesCS3B02G312300, TraesCS3D02G278400, TraesCS3A02G278100 etc. were expressed in the grain_z71 stage. 

### 2.7. Transcript Abundance under Biotic Stress

To study the role of *TaPERK* genes under biotic stress, the expression patterns against two fungal pathogens (Bgt and Pst) were analyzed. A significant correlation (R^2^ = 0.98) was observed in replicates of the expression data (Figure 6B). The genes with FPKM values less than 0.5 were not considered for expression analysis. A total of 18 genes were found affected (Figure 6D). Approximately 50% of genes were upregulated after Bgt infestation while 50% were upregulated after Pst infestation. This shows that the genes are pathogen-specific. For instance, TraesCS3B02G325100, TraesCS3B02G179300, TraesCS1D02G339600 etc. were upregulated after Bgt attack while TraesCS3A02G003900, TraesCS3A02G278100, TraesCS3B02G312300 etc. were upregulated after Pst attack. TraesCS3A02G290300 gene was found to be upregulated after the attack of both fungi. Moreover, a few genes were either late or early responsive. For example, TraesCS1A02G127900 was upregulated after 24 h of Pst attack, while TraesCS3D02G278400 was upregulated after 48 h of Bgt attack. 

### 2.8. Transcript Abundance under Abiotic Stress Conditions

**Figure 7:**
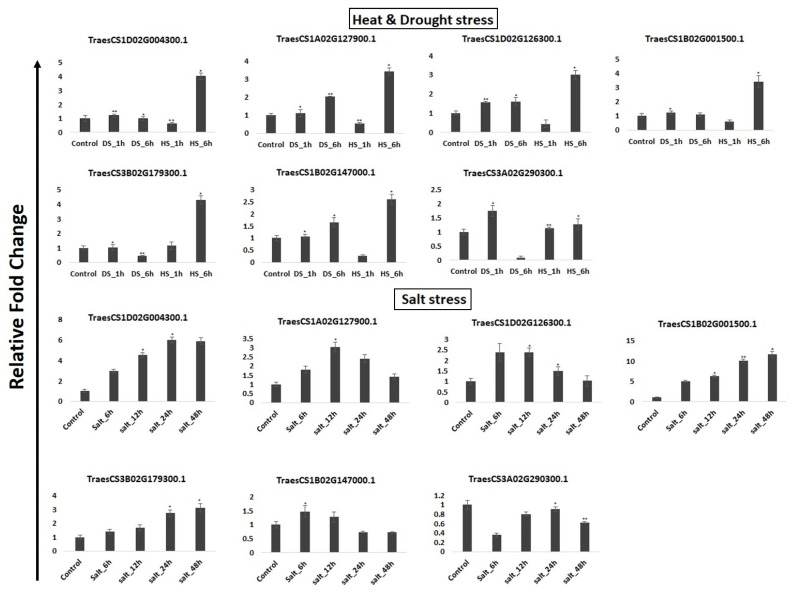
Quantitative expression validation of seven randomly chosen *TaPERK* genes under HS (heat stress) and DS (drought stress) for 1 and 6 h, as well as salt stress for 6, 12, 24, and 48 h. The data are represented as the mean SD (*n* = 3) and a significant difference in comparison to the control was determined using two-way ANOVA followed by Tukey’s post hoc test. * denotes a statistically significant difference at *p* < 0.05; ** indicates a statistically significant difference at *p* < 0.01. The standard error is shown on the bar.

Under abiotic stress, an expression analysis was performed under heat (HS), drought (DS), and salt stress conditions using the high throughput RNA seq data, and qRT-PCR. A total of 18 *TaPERK* genes were affected after the HS and DS stress conditions (Figure 6E). The majority of *TaPERK* genes showed similar expression after 1 and 6 h of DS, while upregulated only after HS 6 h, for instance, TraesCS1D02G004300 and TraesCS1B02G001500. Only TraesCS3B02G325100 was found to be upregulated after 1 and 6 h of HS and DS, except for DS 6h where it was downregulated. A few genes such as TraesCS3A02G290300, TraesCS3D02G290100 etc. showed upregulation only at DS_1h. To further validate the expression data, qRT-PCR of randomly selected seven genes named TraesCS1D02G004300, TraesCS1A02G127900, TraesCS1B02G001500, TraesCS1D02G126300, TraesCS1B02G147000, TraesCS3B02G179300 and TraesCS3A02G290300 was performed using the gene-specific primers (Figure 7, Table 2). These genes varied in expression value but showed similar expression patterns. For instance, TraesCS1A02G127900 and TraesCS1B02G147000 genes were upregulated after each hour of heat and drought stress except HS_1 h in the qRT-PCR experiment, which was found consistent with transcriptomic data.

Under salt stress, 21 *TaPERK* genes exhibited differential expression patterns (Figure 6F). Almost half of the genes showed upregulation in the initial hours (6 h and 12 h) of salt stress, e.g., TraesCS3A02G003900, TraesCS1D02G126300, etc. Meanwhile, half of the genes were upregulated in the later hours (24 and 48 h) of salt stress, e.g., TraesCS3B02G179300, TraesCS1B02G001500, etc. Furthermore, TraesCSU02G104700, TraesCS7A02G231900 and TraesCS7B02G130400 genes were downregulated after each hour of salt stress. TraesCS3B02G179300, TraesCS1B02G001500, TraesCS1D02G004300, TraesCS1B02G350100 and TraesCS3D02G160000 were found to be upregulated after each hour of salt stress. The qRT-PCR under different salt stress intervals (6, 12, 24, 48 h) was performed to validate the transcriptome data. The expression value of each gene varied with the transcriptomic data, but the *TaPERK* genes followed a similar expression pattern. The qRT PCR results were mostly statistically significant at *p* ≤ 0.05, and were consistent with the expression pattern obtained from the RNA seq data (Figure 7).

### 2.9. miRNAs Interaction Network

**Figure 8:**
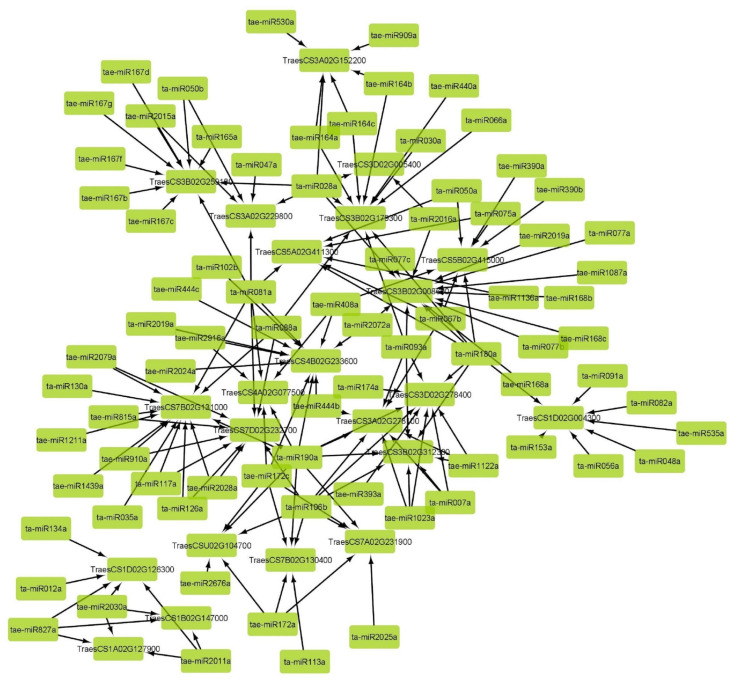
miRNA interaction network. The networks of interactions between *TaPERK* genes and the known *T. aestivum* miRNAs are shown. The psRNATarget program predicted the interaction, and the Cytoscape software created the network. The arrows are aiming towards *TaPERK* transcripts via miRNAs.

Out of 607 known *T. aestivum* miRNAs, 78 interacted with 22 *TaPERK* transcripts, with 60 miRNAs acting via cleavage and 11 via translational inhibition (Figure 8, Table S4). Seven of these miRNAs worked through both the cleavage and translation processes. For example, ta-miR093a acted on TraesCS3A02G278100 by cleavage while on TraesCS3B02G008600 via translational inhibition. Whereas ta-miR180a targeted TraesCS3B02G008600 and TraesCS1D02G004300 via cleavage and translational inhibition respectively. Furthermore, multiple miRNAs targeted the same *TaPERK* transcripts, such as TraesCS3B02G008600, which was targeted by 13 miRNAs via the process of cleavage or translation. Similarly, single miRNA also targets multiple transcripts such as tae-miR2011a. It was discovered that more than one miRNA also targeted the same transcript at the same place, for example, ta-miR2019a and ta-miR2072a target TraesCS4B02G233600 at the same site. Furthermore, certain miRNAs targeted the transcript at two separate sites, such as ta-miR050a and ta-miR081a, which target TraesCS5B02G415000 and TraesCS5A02G411300, respectively. Also, it was observed that a single transcript TraesCS7D02G232700 was targeted by two different miRNAs, i.e., tae-miR2079a and ta-miR088a by the cleavage and translation process, respectively. 

## 3. Discussion

Several studies have explored the role of *PERK* genes in the growth and development and stress responses of various plant species [11,13,14], but none describing the PERK genes and their functions in *T. aestivum* has been carried out to date. *T. aestivum* is an important cereal crop which is cultivated and consumed worldwide [20]. Therefore, an attempt has been made to characterize the *PERK* gene family in this plant. In the current study, 30 *TaPERK* genes were identified from the whole genome of *T. aestivum* and analyzed for their physicochemical properties, domain and motif, expression during tissue developmental stages and under various stress conditions, and miRNA interaction network development. The number of *TaPERK* genes was found to be relatively higher than the genes reported in other plant species. A total of 15, 25, 33 and eight *PERK* genes were reported from the genome of *A. thaliana*, *B. rapa, G. hirsutum*, and *O. sativa* [9,11,12,17]. The number of the genes in the allohexaploid genome of *T. aestivum* suggested the direct connection to the ploidy level and genome size. 

A phylogenetic analysis revealed the formation of four different clades (I-IV), with clade I being the largest with a maximum number of proteins. The study was consistent for the formation of I-IV clades in *B. rapa* and *G. hirsutum* [9,17]. The clade I–III included the PERK proteins from each *A. thaliana* and *O. sativa*, and *T. aestivum*, while clade IV lacked the AtPERK proteins. The studies done in *B. rapa* and *G. hirsutum* also showed the absence of AtPERKs from the clade IV, which also supported our results [9,17]. This suggests that PERK proteins of clade IV might acquire new functions via divergence. It was also observed that orthologs of TaPERK proteins were in close proximity with AtPERK proteins, which suggested their conserved functional role. For instance, At4G32710 known as AtPERK1 expressed more in reproductive organs such as bolts, buds, and siliques found nearby to the TraesCS1D02G339600 in the same clade, which suggests that it might be involved in the growth of reproductive organs. 

The analyses of physicochemical properties of TaPERK proteins predicted the variable length and molecular weight, which were consistent with the results in *B. rapa* and *G. hirsutum* [9,13,17]. The majority of genes with more than 7 pI suggested their basic nature. The occurrence of a negative GRAVY score indicated the overall hydrophilic nature of these proteins which might be due to their larger cytoplasmic kinase domain. The subcellular localization of the majority of proteins was predicted to be plasma membrane, which was also experimentally proved by the biolistic bombardment of onion epidermal cells for PERK1 of *B. napus* [11]. Further, a few proteins named TraesCS1B02G001500, TraesCS1B02G350100, TraesCS1D02G339600, TraesCS3D02G005400 and TraesCS3D02G290100 lacked a transmembrane region, which suggested these proteins might be receptor-like cytoplasmic kinases. However, all the PERK proteins of *A. thaliana* and *B. rapa* exhibited the occurrence of the transmembrane domain [9,13]. The signal peptide was also found to be absent in AtPERK like BnPERK1 which further supported our finding [13]. 

Furthermore, all the PERK proteins exhibited similar patterns of the domain and motif distribution (Figure 4B–D). Like AtPERK members [8,11,13], the proline-rich extracellular region of TaPERKs also contained consecutive prolines at various locations, along with several serine-proline or proline-serine repeats. The YXY motif was also found conserved in numerous TaPERKs, which was not reported in earlier studies. The occurrence of serine-proline repeats and YXY motifs in the N-terminus region are the characteristic feature of the EXT motif [10]. The EXT motif is known to act as the putative sensor for the EXT-pectin glyco-network and maintains cell wall integrity [10]. The occurrence of these motifs in TaPERK proteins suggested that these proteins might also sense EXT-pectin glycol-network for cell wall maintenance. Also, the sequence logos of the conserved PERK amino acid residues showed that the PERK proteins are evolutionarily conserved among the *A. thaliana* and *O. sativa* due to the presence of conserved amino acid residues also reported in *B. rapa* and *G. hirsutum* [9,17].

The *TaPERK* promoter sequences comprise a variety of anticipated *cis*-elements that are specialized for plant growth and development, hormone-responsive, light and stress responses. Transcription is controlled by transcription factors binding to upstream *cis*-acting regulatory elements. Many studies have demonstrated the importance of light in plant growth and development processes [21] which have also been found in some *TaPERK* genes of *T. aestivum* such as *TraesCS3D02G005400, TraesCS3D02G160000, TraesCS3D02G278400, TraesCS3D02G290100* etc., which suggested their role in growth and development processes. The majority of *TaPERK* genes contain characteristics that are typical of genes involved in growth and stress responses. For example, in Arabidopsis, *AtPERK4* of *A. thaliana* displayed ABA-responsive elements for the root-tip development [14]. Similarly, *TaPERK* named *TraesCS1A02127900* consisted of ABA-responsive element and was found to be significantly expressed in all the stages of root development, which indicated its similar role to the orthologous *AtPERK4* of *A. thaliana*. The other RLK gene families in wheat have also been previously reported to contain similar *cis*-elements in their promoters and were functionally relevant [19].

In *A. thaliana*, *AtPERK* members are implicated in several developmental pathways, including the root, rosette leaf, stem, and pollen tissues developments [8,16]. The expression levels of several *TaPERK* genes were clearly varied across the five tissues (root, stem, leaf, spike, and grain) of *T. aestivum*, and some of these genes were significantly and specifically expressed in vegetative and reproductive organs. There was a significant correlation between these findings and those in *A. thaliana* [13,16], *G. hirsutum* [17], and *B. rapa* [9]. The majority of *TaPERK* genes showed higher expression in root, stem, and spike such as, *TraesCS3B02G312300, TraesCS3D02G278400, TraesCS3A02G278100, TraesCS1D02G126300, TraesCS1B02G147000* etc. Similarly, a few AtPERK genes like *PERK8* and *PERK13* were shown to be highly expressed in expanding root hairs, and *PERK5* and *PERK12* genes in pollen tube growth [8,9]. The *PERK5* and *PERK12* genes were also clustered in the same clade with *TaPERK* genes expressing in root and stem mentioned above, which suggested their functional conservation. The results were also found to be similar to PERK genes of *B. rapa* and *G. hirsutum* which suggested that *TaPERK* genes might be involved in plant developmental processes. 

Furthermore, *TaPERK* genes expression was observed to be affected by the attack of fungal pathogens and various abiotic stresses like heat, drought, and salt. Similarly, the *PERK1* gene of *A. thaliana* was functionally validated for its role against wounds induced by mechanical stress and *Sclerotinia sclerotiorum* fungi [11]. The effect of heat, drought, salt, and cold on *PERK* genes of *G. hirsutum* has also been observed and found consistent with our result [17]. Thus, from this, we can conclude that *TaPERK* genes also play a role in defense response. 

The miRNA interaction analysis suggested the regulation of *TaPERK* genes through the RNAi pathway. Numerous miRNAs are known to regulate growth, development, and adaptive response against abiotic stresses by controlling target genes either at the posttranscriptional or translation level of protein synthesis [22,23]. For instance, ta-miR2072a and tae-miR2011a were found to be responsive to heat stress and powdery mildew [24]. These miRNAs target *TraesCS3B02G008600, TraesCS1A02G127900, TraesCS1B02G147000* genes etc., which were significantly upregulated during HS_6h and Bgt infestation. The results suggested that these genes might also be responsive to heat and powdery mildew stress conditions. Further, tae-miR408 has been reported to play a role in plant adaptations to Pi starvation and salt stress conditions via mediating Pi acquisition under low-Pi stress and altering the ABA signaling pathway as well as osmoprotectants biosynthesis [23]. The taemiR408 targets *TraesCS4B02G233600, TraesCS4A02G077500* might be involved in Pi acquisition under salt stress conditions. Further, the tae-mir827a role has been reported in the phosphorous deficient and surplus conditions which target *TraesCS1D02G126300, TraesCS1A02G127900*, and *TraesCS1B02G147000* suggesting their role in maintaining phosphorous metal homeostasis [25]. 

Therefore, coupled with these findings, the study suggested the functional role of *TaPERK* genes in physiological processes and stress responses, which would pave the way for functional characterization in future studies. 

## 4. Materials and Methods

### 4.1. Identification and Chromosomal Distribution of TaPERK Genes in T. aestivum

To identify the *TaPERK* genes in the genome of *T. aestivum*, the bidirectional BLAST hit approach at e-value 10^−10^ was adopted. The arabidopsis PERK protein sequences [11,13] were used as queries against the protein model sequences of *T. aestivum* downloaded from the IWGSC (IWGSC RefSeq assembly v2.0) (http://wheat-urgi.versailles.inra.fr/Seq-Repository/Genes-annotations, accessed on 25 February 2019, http://www.wheatgenome.org/, accessed on 25 February 2019) and the Ensembl Plant (http://plants.ensembl.org/index.html, accessed on 7 August 2021) servers. To validate the retrieved putative PERK sequences, the presence of proline-rich region and kinase domain was analyzed via the multiple sequence alignments and by searching at the SMART server [26,27], respectively.

Predictions of *TaPERK* gene locations on the chromosome of *T. aestivum* were made by mapping each gene sequence on their respective chromosome sequence, downloaded from the Ensembl Plant server. The nomenclature of genes was determined using the recommended rules of the wheat gene symbolization method (http://wheat.pw.usda.gov/ggpages/wgc/98/Intro.htm, accessed on 6 October 2021). The homeologs of each PERK gene were predicted by performing a bidirectional BLAST hit method at e-value 10^−10^. Sequences with ≥90% similarity and localized on different sub-genomes were designated as homeologous as described in previous studies [19,28,29].

### 4.2. Phylogenetic and Synteny Analysis 

The multiple sequence alignment of full-length PERK protein sequences of *A. thaliana, O. sativa and, T. aestivum* was carried out using the MUSCLE algorithm. The phylogenetic tree was then constructed using the MEGA X software by Neighbor-Joining method, with the bootstrap value set to 1000 [30]. The syntenic relationship among the *PERK* sequences of *A. thaliana, O. sativa, and T. aestivum* was performed using the Circos 0.69-6 software [31]. 

### 4.3. Gene and Protein Structure Analyses 

The *TaPERK* gene structure was analyzed in terms of exon-intron organization and intron phases. For this, the respective coding (CDS) and genomic sequences were aligned for each *TaPERKs* and representation has been done using the Gene Structure Display Server (GSDS 2.0) [32]. Further, the various *cis*-regulatory elements were predicted in the 2 Kb upstream region of each *TaPERK* gene using the online PlantCARE program [33]. The pictorial representation of promoter elements was done using the TBtool (v0.6679) software [34]. 

The physicochemical properties (molecular weight, pI, protein length), protein localization, transmembrane helices, GRAVY score, and signal peptide were predicted using the ExPASy, CELLO v.2.5, TMHMM 2.0 version, Sequence Manipulation Suite, SignalP servers, respectively [35,36,37,38]. To predict the conserved amino acid residues, multiple sequence alignment was performed using the MultAlin server. The WebLogo3 (http://weblogo.threeplusone.com/create.cgi, accessed on 27 December 2021) and GeneDoc were used for the representation [26,39]. Further, to predict the conserved signature motif of TaPERK proteins, and the Multiple Expectation Maximization for Motif Elicitation version 5.1.1 was used with the following parameters: the number of motifs; 15, optimum width of the motif; 6 to 50, rest all parameters were set to default [40]. 

### 4.4. Expression Pattern Analysis of TaPERK Genes during Tissue Development Stages 

High throughput RNA-seq data, available at https://urgi.versailles.inra.fr/files/RNASeqWheat, accessed on 24 February 2019, was used for expression profiling of *TaPERK* genes under tissue developmental stages. The RNA seq data were generated in two biological replicates from five different tissues (root, leaf, stem, spike, and grain) and three development stages of the wheat plant [41,42]. The fragments per kilobase of transcripts per million mapped reads (FPKM) value was determined through RNA-Seq by Expectation-Maximization (RSEM) with the help of the Trinity pipeline [43]. The heat maps were generated with the help of Hierarchical Clustering Explorer 3.5 (http://www.cs.umd.edu/hcil/hce/, accessed on 4 January 2022). The expression data was revalidated at the Expression Atlas server [44].

### 4.5. Expression Profiling of TaPERK Genes under Biotic and Abiotic Stresses 

To study the effect of two fungal pathogens viz. *Puccinia striiformis* (Pst) and *Blumeria graminis* (Bgt) on the expression pattern of *TaPERK genes*, the RNA seq data has been used. RNA seq data generated by Zhang et al. in three biological replicates after 24, 48, and 72 h of infestation of these two fungal pathogens were used [45]. The expression value of *TaPERK* genes were calculated in terms of FPKM using the Trinity package [43]. Further, the expression profiling of *TaPERK* genes under heat (HS), drought (DS), and salinity stress was analyzed using the RNA seq data reported by Liu et al. For the heat and drought stresses, the data were generated in two biological replicates from the leaves tissue after 1 and 6 h of treatment [46]. However, for the salinity stress (150mM NaCl) the data were developed from the root tissue after 6, 12, 24, and 48 h of treatments, separately [47]. For the representation of expression pattern, the heatmaps were constructed using the HCE (Hierarchical Clustering Explorer 3.5) with the parameters as Euclidean distance method and hierarchical clustering [48]. 

### 4.6. RNA Extraction and Real-Time Quantitative PCR Analysis for Gene Expression 

Seeds of *T. aestivum cv* were surface sterilized and after seven days of growth, the seedlings were subjected to heat (40 °C for 1 and 6 h), drought or osmotic (20% PEG for 1 and 6 h), and salinity stress (150 mM NaCl for 6, 12, 24 and 48 h). Root and shoot tissue samples were collected after the stress treatment and RNA was isolated using the SpectrumTM Plant Total RNA kit (Sigma, Burlington, MA, USA). The RNA was treated for the removal of DNA contamination using the TURBO DNA-free™ Kit (Invitrogen, Waltham, MA, USA). Further, the quality and integrity of the isolated RNA were checked by gel electrophoresis and concentration was measured using the Nanodrop spectrophotometer. The first-strand cDNA was synthesized from the 1 ug RNA sample using the Superscript III First Strand Synthesis Supermix (Invitrogen, USA). For the qRT-PCR analysis, seven *TaPERK* genes were randomly selected from each phylogenetic clade. The gene-specific primers were designed from OligoCalc software [49]. The QuantiFast^®^ SYBR^®^ Green PCR Kit (Qiagen, Hilden, Germany) was used for qRT PCR on Bio-Rad CFX96 Real-Time PCR detection system following the method established in the laboratory [50]. The experiments were performed in triplicate and for normalization. The internal reference gene ADP-ribosylation factor (TaARF1, AB050957.1) was used and calculations were made using the delta-delta CT-method (2^−ΔΔCT^) [51].

### 4.7. miRNA Target Prediction and Interaction Network Development

To predict the interacting miRNA of *TaPERK* genes, a total of 607 known miRNAs of *T. aestivum* [52] were used for interaction analyses using the psRNATarget (http://plantgrn.noble.org/psRNATarget/, accessed on 10 January 2022) server [53]. The representation of the interaction network was developed using the Cytoscape software [54]. 

### 4.8. Statistical Analysis

To check the reproducibility of high throughput RNA seq data generated from tissue developmental stages and biotic and abiotic stress conditions, a correlation analysis was performed using Microsoft Office Excel. The qRT PCR data were statistically analyzed by two-way Analysis of Variance (ANOVA) and Student’s *t*-test at the probability level of 5% using the SPSS software. For the determination of significant mean difference (*p* < 0.05) among the treatments the post hoc Tukey’s test was used.

## 5. Conclusions

The present study provides a comprehensive analysis of the *TaPERK* gene family in *T. aestivum*. Phylogeny and synteny analyses of *PERK* genes from different species indicated the functional conservation and close evolutionary relationship among them. The analysis of PERK gene expression profiling under tissue developmental stages suggested their involvement in the growth and development of the plant and environmental adaptations against different stress conditions. The study is in agreement with previous studies on various plant species. Further, the members of this gene family were also found to be controlled by various miRNAs. These findings provide a basis for the understanding of the potential functional roles of *TaPERK* genes in *T. aestivum*. The potential regulatory mechanisms of *TaPERK* genes need to be further characterized in plants in future studies.

## Figures and Tables

**Table 1:** Various characteristic features of TaPERK proteins.

Gene Names	Gene IDs	Protein Length (AA)	Chromosome	Mol. Wt (kDa)	Subcellular Localization	Transmembrane
*TaPERK1-1A*	TraesCS1A02G127900	658	1A	69.45	Plasma membrane	1
*TaPERK1-1B*	TraesCS1B02G147000	617	1B	65.24	Plasma membrane	1
*TaPERK1-1D*	TraesCS1D02G126300	653	1D	69.08	Plasma membrane	1
*TaPERK2-1B*	TraesCS1B02G001500	359	1B	39.94	Plasma membrane	0
*TaPERK2-1D*	TraesCS1D02G004300	656	1D	68.94	Plasma membrane	1
*TaPERK3-1B*	TraesCS1B02G350100	451	1B	48.3	Plasma membrane	0
*TaPERK3-1D*	TraesCS1D02G339600	451	1D	48.39	Plasma membrane	0
*TaPERK4-3A*	TraesCS3A02G003900	687	3A	72.43	Plasma membrane	1
*TaPERK4-3B*	TraesCS3B02G008600	686	3B	71.89	Plasma membrane	1
*TaPERK4-3D*	TraesCS3D02G005400	341	3D	37.88	Cytoplasmic	0
*TaPERK5-3A*	TraesCS3A02G152200	630	3A	67.44	Plasma membrane	1
*TaPERK5-3B*	TraesCS3B02G179300	631	3B	67.47	Plasma membrane	1
*TaPERK5-3D*	TraesCS3D02G160000	632	3D	67.5	Plasma membrane	1
*TaPERK6-3A*	TraesCS3A02G229800	720	3A	74.98	Plasma membrane	1
*TaPERK6-3B*	TraesCS3B02G259100	698	3B	72.99	Plasma membrane	1
*TaPERK7-3A*	TraesCS3A02G278100	675	3A	72.43	Plasma membrane	1
*TaPERK7-3B*	TraesCS3B02G312300	677	3B	72.61	Plasma membrane	1
*TaPERK7-3D*	TraesCS3D02G278400	676	3D	71.26	Plasma membrane	1
*TaPERK8-3A*	TraesCS3A02G290300	721	3A	75.49	Plasma membrane	1
*TaPERK8-3B*	TraesCS3B02G325100	811	3B	84.78	Plasma membrane	1
*TaPERK8-3D*	TraesCS3D02G290100	438	3D	47.15	Cytoplasmic	0
*TaPERK9-4A*	TraesCS4A02G077500	621	4A	64.5	Plasma membrane	1
*TaPERK9-4B*	TraesCS4B02G233600	618	4B	64.46	Plasma membrane	1
*TaPERK10-5A*	TraesCS5A02G411300	573	5A	60.34	Plasma membrane	1
*TaPERK10-5B*	TraesCS5B02G415000	613	5B	64.69	Plasma membrane	2
*TaPERK11-7A*	TraesCS7A02G231900	643	7A	66.57	Plasma membrane	1
*TaPERK11-7B*	TraesCS7B02G130400	764	7B	79.88	Plasma membrane	1
*TaPERK11-U*	TraesCSU02G104700	734	Un	76.57	Plasma membrane	1
*TaPERK12-7B*	TraesCS7B02G131000	753	7B	78.66	Plasma membrane	1
*TaPERK12-7D*	TraesCS7D02G232700	751	7D	78.21	Plasma membrane	1

**Table 2:** The gene-specific primers for the qRT-PCR experiment.

Gene Name	Primer Sequences (5′ to 3′)
TraesCS1D02G126300_F	ACGCCAGGCCACAACAACCCGTCG
TraesCS1D02G126300_R	GAGGCTGGCGGCGGCGCTTCTT
TraesCS1A02G127900_F	TGGCGGGGGAAGATCGCCTTC
TraesCS1A02G127900_R	CCGGAGGCAGCAGAGGCACAC
TraesCS1D02G004300_F	TTCCACTTCCACGCCGCCTCCAAC
TraesCS1D02G004300_R	AGGCGGCGATGGCTTGGCGTG
TraesCS1B02G001500_F	CCGATGTGTTCTCTTTTGGTGTGGTACT
TraesCS1B02G001500_R	ATGCAGGCGGCTGCAGATTCAATCATA
TraesCS3B02G179300_F	TGATCGCGCTGCTGCTCGCCAGC
TraesCS3B02G179300_R	GAGGGGCATTATGCTGCCACCCATAT
TraesCS1B02G147000_F	GCCCTTGGTGCTGCTAAGGGTTTG
TraesCS1B02G147000_R	CCCAAAAGTGCCCATTACTCTTGTTGAC
TraesCS3A02G290300_F	ACCCCCGGTGAATCCTCCTCC
TraesCS3A02G290300_R	TGAACGTGGCACGTCGGTCGG
TaARF_F	TGATAGGGAACGTGTTGTTGAGGC
TaARF_R	AGCCAGTCAAGACCCTCGTACAAC

## Data Availability

All the data used in this study is freely available in the databases/repositories. Link and accession number has been mentioned in the manuscript.

## Supplementary Materials

The following supporting information can be downloaded at: https://www.mdpi.com/article/10.3390/life12070941/s1, Table S1: List of TaPERK genes identified in *A. thaliana*, *O. sativa* and *T. aestivum*; Table S2: Analysis of various characteristic features of *TaPERK* genes and proteins of *T. aestivum* L.; Table S3: Various *cis*-acting regulatory elements in the 2 Kb upstream region of *TaPERK* genes.; Table S4: List of interacting miRNA as target mimic or direct target of *TaPERK* genes; Figure S1: Multiple sequence alignment. The alignment of the extracellular region of TaPERK proteins shows conserved proline residues are marked with red color, EXT motif sequence, i.e., Ser-pro residues marked with green color while pro-ser residues marked with orange color. The alignment is depicting the conserved residues of PERK proteins in *T. aestivum*.; Figure S2: The figure represents the conserved motif sequence logos generated from MEME suite using full-length TaPERK proteins.
