# Supplementary figures and images for: Insight into the Roles of Proline-Rich Extensin-like Receptor Protein Kinases of Bread Wheat (Triticum aestivum L.)
Source: Life (Basel). 2022 Jun 23;12(7):941. doi: 10.3390/life12070941 (PMC9323123; doi:10.3390/life12070941)

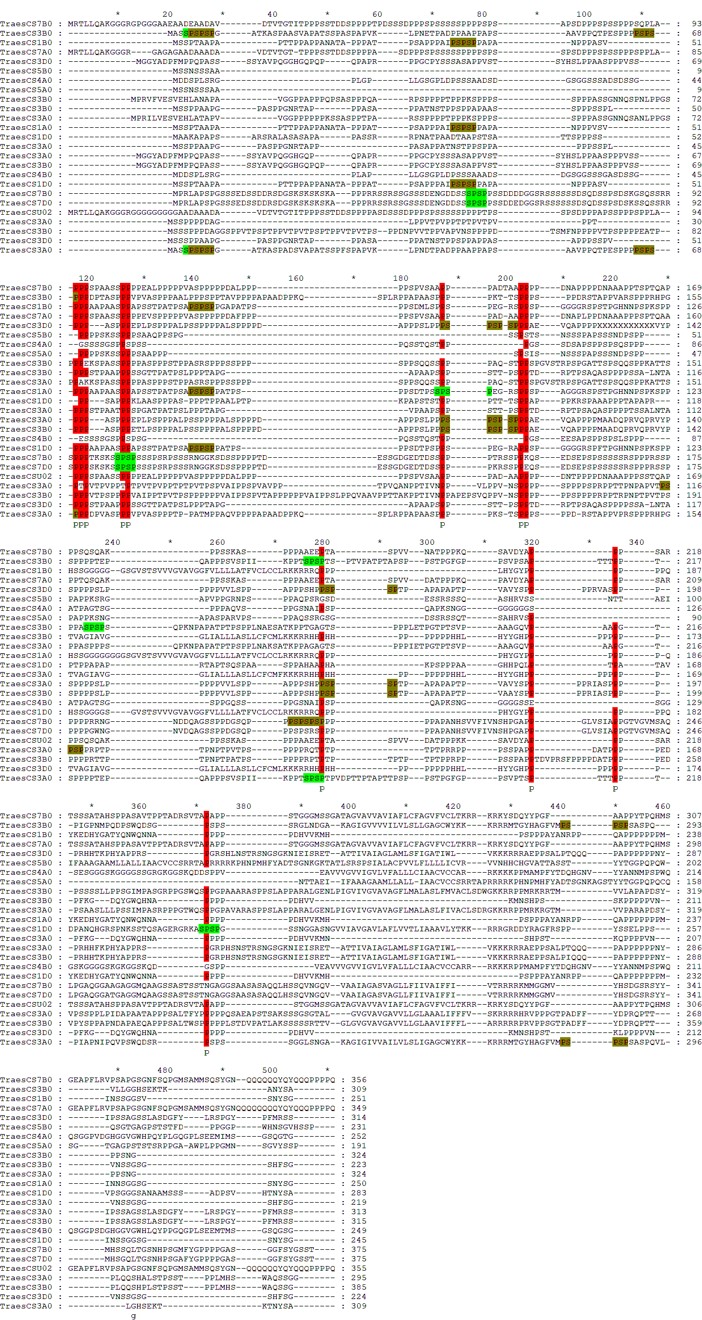

Supplement: Supplementary file 1 [file life-12-00941-s001.zip › Figure S1.jpg]

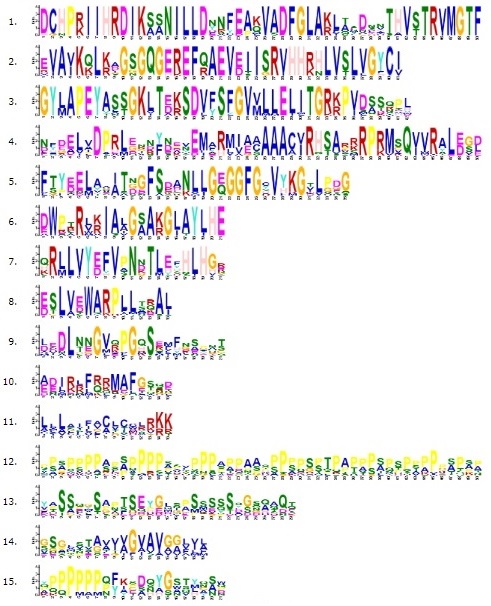

Supplement: Supplementary file 1 [file life-12-00941-s001.zip › Figure S2.jpg]
